# Correction: Hdac3 is an epigenetic inhibitor of the cytotoxicity program in CD8 T cells

**DOI:** 10.1084/jem.2019145305152020c

**Published:** 2020-05-22

**Authors:** Rong En Tay, Olamide Olawoyin, Paloma Cejas, Yingtian Xie, Clifford A. Meyer, Yoshinaga Ito, Qing Yu Weng, David E. Fisher, Henry W. Long, Myles Brown, Hye-Jung Kim, Kai W. Wucherpfennig

Vol. 217, No. 7 | 10.1084/jem.20191453 | May 6, 2020

The authors regret that in the original version of this article, Yoshinaga Ito was inadvertently omitted from the author list. The corrected author list appears above.

All versions of this article have been corrected. The error occurs in PDFs downloaded before May 14, 2020.

